# Topical Review: Basic Psychological Needs in Adolescents with Chronic Pain—A Self-Determination Perspective

**DOI:** 10.1155/2019/8629581

**Published:** 2019-01-06

**Authors:** Annina Riggenbach, Liesbet Goubert, Stijn Van Petegem, Rémy Amouroux

**Affiliations:** ^1^Family and Development Research Center, Institute of Psychology, University of Lausanne, Switzerland; ^2^Department of Experimental-Clinical and Health Psychology, Ghent Health Psychology Lab, Ghent University, Belgium

## Abstract

This topical review outlines the resilience pathway to adaptive functioning in pediatric pain within a developmental perspective. Self-Determination Theory proposes that the satisfaction of one's basic psychological needs (for autonomy, relatedness, and competence) is crucial for understanding human flourishing and healthy development. However, the role of the basic psychological needs received little attention in a pediatric-pain population. Yet, we propose that need satisfaction may be a resilience factor and need frustration a risk factor, for living with chronic pain. In this topical review, we first discuss two major models that have been developed to understand pain-related disability: the fear-avoidance model of pain and the ecological resilience-risk model in pediatric chronic pain. Both models have been used with children and adolescents but do not include a developmental perspective. Therefore, we introduce Self-Determination Theory and highlight the potentially moderating and mediating role of the basic needs on pain-related disability in children and adolescents. Taken together, we believe that Self-Determination Theory is compatible with the fear-avoidance model of pain and the ecological resilience-risk model in pediatric chronic pain and may deepen our understanding of why some adolescents are able to live adaptively in spite of chronic pain.

## 1. Introduction

**Figure 1:**
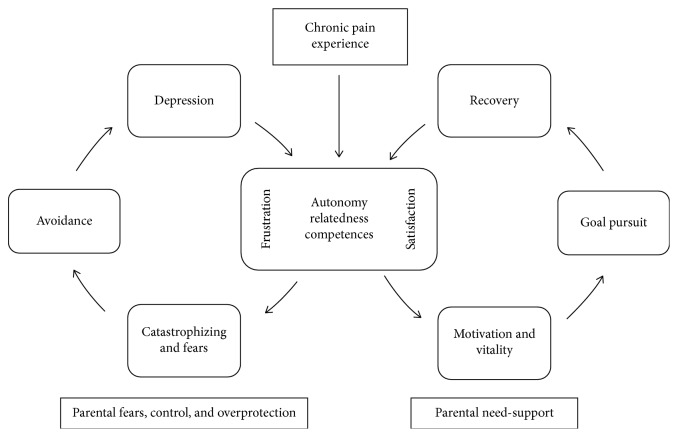
A Developmental Goal Pursuit Model of Chronic Pain. Need satisfaction facilitates goal pursuit while need frustration leads to avoidant behaviors. A need-supportive context improves needs satisfaction.

Children and adolescents frequently experience pain [1]. About 25% of young people report persistent pain (>3 months) [2] and 8% of them describe their pain as severe and disabling [3, 4]. Thus, chronic pain can significantly disrupt the development of children and adolescents and hamper the pursuit of personal goals [5–7]. Paradoxically, at the same time, many youngsters with chronic pain are able to live adaptively and pursue personal goals, in spite of experiencing chronic pain [3, 6, 8, 9]. An important question to consider, then, is *why* some children and adolescents are able to live adaptively and to continue pursuing their personal goals in spite of their chronic condition, while others are not. The aim of this topical review is to outline the resilience pathway to adaptive functioning in pediatric pain within a developmental perspective. Specifically, we draw upon Self-Determination Theory (SDT [10]) to argue that the satisfaction of one's basic psychological needs and their contextual support are important resources for adolescents to live adaptively with chronic pain (Figure 1). Thereby, we consider various types of chronic pain, as evidence has shown that the emotional, behavioral, and psychosocial factors influencing functional disability are generally similar across different types of pain [11].

For many years, the Fear-Avoidance Model of pain (FAM) [12] partially explains the dynamics involved in chronic pain and has been one of the principal guiding frameworks for research on chronic pain in both adults [13] and children [14, 15]. This model describes risk mechanisms for disability at emotional (e.g., fear), cognitive (e.g., catastrophizing), and behavioral (e.g., avoidance) levels and has been extended to the interpersonal context [16]. First created in the context of chronic low back pain [17], the relevance of FAM has been generalized to other types of pain, such as headache [18], abdominal pain [19], neuropathic pain, and complex regional pain syndrome [20]. Recently, resilience mechanisms leading to recovery and adaptive living (as opposed to fear and avoidance) have received increasing attention. In this context, restoring and pursuing personal goals (i.e., personally valued goals, such as school, professional, sporting, or social goals) has been shown to be an “antidote” to the fear-avoidant downward spiral among both adults and children [6, 21–23]. The ecological resilience-risk model of pediatric chronic pain [24] aimed at deepening the understanding of resilience mechanisms in chronic pain, identifying resilience factors (such as optimism, positive emotions, and positive social interactions) that may foster adaptive living with chronic pain. In this context, personal goal pursuit would be considered as resilient mechanisms for adaptive living while fears, catastrophizing, and depression would be considered as risk factors.

However, although some research highlighted the importance of the development in pain-related outcomes [25, 26], none of those models explicitly considered developmental variables in their perspective of risks and resilience. In that respect, SDT [10, 27] may shed light on developmental processes related to risk and resilience in the context of chronic pain. SDT posits that personal goal pursuit, and optimal development in general, is facilitated through the satisfaction of one's basic psychological needs (for autonomy, competence, and relatedness) and through the contextual support of these needs [28, 29]. In addition, SDT provides insight into the conditions under which parental involvement brings about positive or negative outcomes on need satisfaction.

This topical review aims to connect concepts coming from different theoretical fields. Specifically, by discussing the potential relation between the basic psychological needs, resilience resources (and more specifically the pursuit of personal goals), and risk factors (described in terms of fear-avoidant mechanisms), we hope to foster our understanding of what makes adaptive living possible among youngsters with chronic pain. We will argue that children's and adolescents' psychological needs may often be frustrated by pain, but also that the contextual support of these needs may function as a resilience resource. Further, we will discuss the influence of parental need support on adolescents' need satisfaction and goal pursuit. After introducing the fear-avoidance model of pain (with its extension on interpersonal mechanisms and goals pursuit) and the ecological resilience-risk model, we will present Self-Determination Theory and the basic psychological needs. Then, we will review the existing literature in pediatric chronic pain through the lens of SDT. Finally, we will provide an outline for future research and discuss clinical implications.

## 2. Fear-Avoidance and Resilience in the Context of Pediatric Chronic Pain

The *Fear-Avoidance Model of pain* [12] describes why and how people with pain may develop a chronic pain syndrome. This model asserts that when perceiving pain as threatening, an individual might have catastrophic thoughts and respond with pain-related fear and avoidance behavior which, in turn, would result in declines in functioning [12]. This, in turn, may lead to a vicious circle where fear leads to more avoidance, more pain, and impairment, as well as an increased risk for persisting pain. This theoretical model has been a major framework for guiding pain research and management in children, adolescents, and adults [13–15, 23]. In other words, fear and avoidance can be considered as risk factors for increasing pain-related disability. The FAM also shows that engaging in important personal goals (e.g., professional, familial, or leisure goals) helps to get out of the downward spiral of fear and avoidance and may lead to recovery and adaptive living [30].

Initially, the FAM only considered individual variables—and not so much interpersonal ones - for defining risks and resources towards living with chronic pain. The *Interpersonal Fear Avoidance Model of pain* (IFAM; [16]) aimed to take into account interpersonal and contextual dynamics as well: indeed, one's response to pain is not only an individual process, as people are embedded in an interpersonal context, which may influence their experiences and reactions to pain. For example, in the context of pediatric pain, parental distress may considerably influence negatively the child's pain-related outcomes [31–35] and the perpetuation of pain-related disability [36, 37]. To illustrate, parents who interpret a child's pain expression through their own catastrophic thoughts and pain-related fears are more likely to engage in maladaptive parenting behaviors and to provide “miscarried” help, such as overprotecting their child, giving special attention, or keeping the child home from school [38–40]. Unfortunately, such miscarried help would further prevent the child from engaging in daily activities [16, 31, 41–43], as well as from pursuing his/her personal goals [44, 45]. In other words, by doing so, overly involved parents would involuntarily perpetuate their child's pain-related disability. It is also important to note that parental responses may also be influenced by the child's own personality and relation to his/her pain [46–48]. For example, children and adolescents who catastrophize about their pain and who engage in maladaptive behaviors (e.g., avoidance) might make it difficult for the parent to encourage engaging in adaptive behaviors.

A third model related to FAM, the *Goal Pursuit Model of Pediatric Chronic Pain* [6, 49] has recently been proposed. This model encompasses the interpersonal context of the IFAM [16] and goal pursuit as proposed in the FAM [23]. This goal pursuit model specifically focuses on child factors (e.g., the motivation to pursue a specific goal, pain-related anxiety, or fears) and parent factors (e.g., overprotective behaviors, anxiety, or fear about their child's pain), as both could hinder or encourage goal pursuit behaviors in youths with chronic pain [6].

Finally, the *ecological risk-resilience model of pediatric chronic pain* [24] describes resources and mechanisms that may lead to recovery and sustainability while living with chronic pain, identifying both individual and interpersonal resilience factors. Resilience is defined as a person's ability to respond effectively to adversity and people's resilience resources are influenced by developmental, social, cultural, and environmental factors [50]. For example, optimism, mindfulness, or positive emotions are considered as individual resilience resources, while positive peer relationships, social connectedness, or parents' and teachers' support are interpersonal resilience resources. In addition, specific resilience mechanisms might be activated when being confronted with pain. Individual resilience mechanisms are, for example, self-efficacy, and psychological flexibility (i.e., responding in an effective and flexible way to adverse events, such as chronic pain [51]). Examples of interpersonal resilience mechanisms are parent's active coping, promotion of behavioral activation, and parents' psychological flexibility (encompassing values-based action, pain acceptance, and emotional acceptance) [52, 53]. By contrast, fears and catastrophizing are individual risk mechanisms, while depression and anxiety are individual risk factors. Parent solicitous responses, parent catastrophizing, and fears are interpersonal risk mechanisms, while parent poor health status constitutes a risk factor [24].

Taken together, researchers increasingly agree that (1) it is important to consider the interpersonal context and (2) the pursuit of personal goals may predict positive outcomes among children and adolescents with chronic pain, potentially serving as a resilience factor. However, the discussed models do not so much incorporate a developmental approach. Yet, doing so may help explain why one would engage either in goal pursuit or avoidant behaviors. Herein, we propose that Self-Determination Theory [54] may help us gain further insight into the factors that facilitate personal goal pursuit among children and adolescents with chronic pain.

## 3. The Added Value of Self-Determination for Understanding Pediatric Chronic Pain

Self-Determination Theory (SDT; [10, 27]) is a macrotheory of human motivation, emotion, and personality and can be situated within the positive psychology movement, as it attempts to explain how to support and enhance personal growth and human flourishing [55]. A key assumption of this theory is the existence of three basic psychological needs—the need for autonomy, competence, and relatedness. These needs are considered to be innate psychological nutriments, as their satisfaction would be essential for psychological growth, subjective well-being, and optimal human functioning, whereas their frustration would lead to maladjustment and the development of psychopathology [29, 56]. When satisfied in their need for *autonomy*, one would experience a sense of volition, personal choice, and psychological freedom in one's actions [29]. Autonomy frustration, by contrast, involves feeling forced or coerced to act in a certain way. Competence satisfaction refers to feeling effective and capable to achieve desired outcomes [57], whereas competence frustration involves feelings of doubt and failure concerning one's efficacy. The need for *relatedness* refers to the experience of intimacy and genuine connection with others [57]. Relatedness frustration involves the experience of relational exclusion and loneliness.  Table 1 provides a summary and brief examples of the three basic psychological needs in the context of chronic pain. A growing body of research shows that regardless of age or cultural background, the satisfaction of these needs contributes to individuals' well-being, social adjustment, and motivation [28, 29, 58]. In addition, research increasingly confirms that need frustration may result in ill-being, maladjustment, and even psychopathology [56, 59–62]. Furthermore, there are studies showing that need frustration is also predictive of a lowered motivation to engage in personally valued goals [63].

Further, SDT underscores the importance of the environment for the satisfaction (vs. frustration) of one's psychological needs, with the parents playing a particularly crucial role throughout childhood and adolescence [10, 64]. In that respect, previous research found that an autonomy-supportive parenting style, which is characterized by perspective-taking, choice provision, and the support of initiative, is predictive of adolescent need satisfaction, which in turn is associated with higher well-being [65, 66] and less problem behavior [67]. Conversely, controlling parenting, which is characterized by the use of coercive, critical, and authoritarian parenting practices and a tendency to enforce the child to act, feel, or think in parent-imposed ways [68], is predictive of the frustration of the child's basic psychological needs, which in turn would relate to more internalizing and externalizing problems [69]. In addition, research drawing upon SDT showed that autonomy-supportive contexts foster children's pursuit of personally valued goals, as such contexts that satisfy children's psychological needs [70].

A limited number of studies applied the SDT framework in the context of health behavior. For example, it was shown that psychological need satisfaction facilitates the successful attainment of health-related goals (e.g., sticking to a diet; Ryan et al.[71]). Only a few studies have been conducted in a pain context. In one study among adolescents from the general population, it was found that teachers' support of autonomy and competence was a protective factor against school absence in youngsters with severe pain problems [9]. In a recent study among partners of adults with chronic pain, Kindt et al. [72, 73] found that higher levels of autonomously motivated help by partners (i.e., feeling free to help versus feeling forced to help) were related to better well-being, need satisfaction, and relationship quality which, in turn, related to better pain-related outcomes [72, 73]. Moreover, a longitudinal study by [74] showed that spousal autonomy support had a positive effect on well-being and need satisfaction of people with chronic musculoskeletal pain, and this is independent of pain intensity [74]. These findings suggest that a Self-Determination perspective is relevant in the context of chronic pain and especially for understanding mechanisms of goal pursuit within an interpersonal context.

## 4. Basic Psychological Needs in the Context of Chronic Pain

For children and adolescents with chronic pain, the presence of pain may challenge need satisfaction and the pursuit of personal goals considerably [75, 76]. Thus, the frustration of their basic needs may explain why chronic pain is predictive of diminished goal pursuit, disability, and functional difficulties, hence playing a mediating role. At the same time, the contextual support of their needs may play a moderating role. That is, it may constitute a resilience factor that explains why some adolescents do well (and continue doing well), even under conditions of high pain. In other words, parents' (and other adults') need support would buffer against the negative effects of chronic pain for adolescents' functioning, as it would positively affect adolescents' need satisfaction.

The mediating role of the psychological needs and the moderating role of a need supportive context are summarized in Figure 1 and are elaborated in the next sections.

### 4.1. Autonomy and Chronic Pain

#### 4.1.1. Autonomy as a Mediator: “My Freedom and My Choices Are Constraint by My Pain”

The development of a sense of autonomy is claimed to be a crucial developmental task for adolescents [77], which can be impaired because of chronic pain [26, 31]. However, the topic of autonomy in adolescents with chronic pain has received little attention until now. First, it is important to clarify the definition and conceptualization of autonomy, as it is a highly debated issue in the developmental literature [78, 79]. In that respect, recent research increasingly underscores the importance of distinguishing between two conceptualizations of autonomy, which has important implications for the question whether autonomy is (always) adaptive for adolescents, or whether offering too much autonomy may imply certain risks. A first definition of autonomy, which is rooted in SDT, conceptualizes autonomy as self-endorsed or *volitional functioning*, which refers to the extent to which one acts upon personally endorsed interests, values, and goals and feels a sense of freedom in his/her choices and actions [29, 79]. The opposite involves controlled or *pressured functioning*, in which case one feels obliged or coerced to act or think in certain ways. For example, in a chronic pain context, an adolescent's autonomy may be observed when his/her choices are congruent with his/her values and interests, for example, when an adolescent wants to become a doctor because he/she likes helping people in difficulty. Importantly, this conceptualization of autonomy should be distinguished from a definition of autonomy as *independence*, which refers to the extent to which one thinks, behaves, and takes decisions without relying on others [80]. The opposite of independence, then, is *dependence* or reliance on others, especially on parents. For instance, when an adolescent with chronic pain needs help from his/her parents to get to school, he/she is (functionally) dependent on the parents. Research among normally developing adolescents has shown that independence (e.g., in family decision-making) gradually increases throughout adolescence [81]. However, research equally found that too much independence, especially when granted too early in adolescence, may relate to maladaptive functioning, including lowered well-being and more problem behavior [82]. Volitional functioning, by contrast, is unequivocally linked to more adaptive functioning, including higher subjective well-being, less behavioral problems, and higher-quality relationship with friends, regardless of adolescents' age [79, 83].

It is important to note that, in adolescence, independence and volitional functioning are not completely orthogonal, that is, there is a modest but positive relation between volitional functioning and independence [79, 83, 84]. In other words, independence may, on average, set the stage and allow for volitional functioning in adolescence. As these two conceptualizations of autonomy are distinct, several combinations are possible. Specifically, an adolescent may choose to decide independently because he/she personally values making the decision by him/herself, which constitutes volitional independence. However, he/she could also act independently because he/she feels pressured to do so. To illustrate, an adolescent might decide by himself about what to study at school because he personally values such independent behavior (i.e., volitional independence). However, he could also decide independently because he feels obliged to figure out things by himself, because his parents believe that he is old enough to decide and take care of his own business (i.e., pressured independence). Similarly, dependency might be volitional or pressured. An adolescent might choose to follow the decision of his/her parents because he/she fully endorses and values their opinion (i.e., volitional dependence) or he/she might follow his/her parents' decision to avoid feelings of guilt or for reasons of loyalty (i.e., controlled dependence; [83]).

This differentiation between autonomy as independence vs. volitional functioning is of crucial importance in the context of chronic pain. That is, chronic pain may easily impair one's independence; nevertheless, one's feelings of volition could remain relatively unaffected, because one may still act upon personally valued choices, even with chronic pain. However, to our knowledge, research on pediatric pain never took into account the distinction between volitional functioning and independence and mostly focused on the implications of pain for one's (in)dependent functioning. As our paper draws upon the SDT framework, we refer to “independence” when discussing studies focusing on adolescents' independent functioning (e.g., independence in decision-making, or distancing from parents) and “autonomy” when we refer to the adolescents' volitional functioning (i.e., acting in congruence with one's personal interests and values).

Previous research among adolescents with chronic pain indicated that these adolescents perceive themselves, and are perceived to be, more dependent on their parents than their peers [5]. That is, they report higher levels of closeness to their parents, show higher levels of dependence on them, and report lower levels of independent decision-making [44, 46, 85]. Thus, these findings suggest that pain may impair adolescents' independence and may prevent them to undertake developmentally appropriate activities, such as attending school and spending time with friends or taking on certain responsibilities (e.g., completing chores at home; [26, 31, 44, 85]. Nevertheless, given the cross-sectional design of past studies, it remains unclear whether the high level of dependence is due to pain, or whether high levels of dependence cause the emergence of pain symptoms [26]; longitudinal research would be needed to shed light on the directionality of effects. Although autonomy might be thwarted by chronic pain, when satisfied, it might have positive effects on pain-related outcomes. Hence, supporting autonomy seems of crucial importance, as is discussed below.

#### 4.1.2. An Autonomy-Supportive Context as a Moderator: “Feeling Supported by My Parents Helps Me to Live with Pain”

In the developmental psychological literature, it is well accepted that an autonomy-supportive context (e.g., from parents, teachers, or peers) yields benefits for adolescents' psychological well-being, growth, and development [66]. Research increasingly suggests that this may also be the case in the context of pediatric pain (Palermo, 2012); [86, 87]. For example, one study showed that teachers' autonomy support (i.e., support of volitional functioning) was a protective factor for adolescents' school functioning, as it related to a higher school frequentation, better school grades, and less bullying experiences in highly impaired children and adolescents with chronic pain [9]. Similarly, in adults with chronic pain [72, 88] and in adults with diabetes [89, 90], autonomy support from a health-care provider and autonomously motivated help from the partner were both protective factors, predicting better adjustment for the person with pain or diabetes. Moreover, both autonomy support and autonomously motivated help had positive effects on treatment adherence and lead to better short-term and long-term treatment outcomes [89–91].

Some research focused on parents' support of independence in children with diabetes. These studies found that health outcomes were more positive, and treatment adherence was better when parents supported independent behaviors in the adolescent [92–94]. However, these studies focused on parental support of the adolescent's independence and not on the adolescent's autonomy as volition. Nevertheless, those findings support the idea that an autonomy-supportive context may be a resource, explaining why some adolescents do well (and even thrive) despite their heightened levels of pain.

From a Self-Determination perspective, parental involvement may be experienced as either autonomy-satisfying or rather as autonomy-frustrating, depending on the way in which it is implemented. For instance, parental overprotection, which is a type of over-involvement, is more likely to be perceived as controlling and hence, autonomy-frustrating, yielding either opposition and resistance (so rather externalizing types of problems) or unhealthy dependence (and rather internalizing problems) [69]. However, when parental involvement is conveyed in an autonomy-supportive way, where parents are sensitive for the adolescents' needs, as well as for their values and goals, parental involvement is less likely to lead to problematic outcomes. In sum, a context of parental, teacher, or health-care autonomy support may foster autonomy satisfaction among adolescents with chronic pain, thus buffering against the negative effects of pain and pain-related disability. Future research is needed to test this in the context of pain.

### 4.2. Competences and Chronic Pain

#### 4.2.1. Competence as a Mediator: “Because of My Pain, I Can't Concentrate at School and My Grades Decline”

Adolescents' feelings of competence are often compromised by chronic pain, because engaging in school and leisure activities and performing physically is often a challenge in the presence of pain [95–99]. Many studies showed that chronic pain is associated with higher school absenteeism and a decline in school grades [3, 97, 100–103]. Surprisingly, however, this decline in school grades is not directly correlated with pain intensity [9, 101]. Moreover, research has shown that adolescents with chronic pain experience higher levels of worrying and more fear of failing than their healthy peers, particularly regarding academic or athletic performances [104, 105]. That is, experiencing pain interference in academic performances brings frustration in the need for competence, which may contribute to avoidance behaviors and patterns of long-term disability [101, 106]. A review by Sinclair et al. [34] found that feelings of incompetence among adolescents with chronic pain were associated with increased activity avoidance and disability and limited the development of adequate strategies to manage pain [34]. Prolonged avoidance and absenteeism might in turn intensify the feeling of incompetence and decrease the motivation to attend school or any other activity, thus creating a downward spiral: chronic pain impairs the satisfaction of the need for competence, which, in turn, triggers avoidant mechanisms, further impacting the well-being of adolescents with chronic pain negatively.

Another aspect of competence is self-efficacy, which, in this context, involves an adolescent's self-perception of being capable of dealing successfully with pain [34]. Self-efficacy in adolescents with chronic pain was positively correlated with quality of life, fewer somatic, behavioral or emotional symptoms, and higher self-esteem [107]. Moreover, higher levels of self-efficacy related to lower levels of experienced pain and other pain-associated symptoms, such as depression and catastrophizing thoughts [107, 108]. In adults with chronic pain as well, feelings of self-efficacy were associated with several positive outcomes, such as better health and physical functioning, and more satisfaction at work and with lower levels of pain intensity, disability, and less depression and fatigue [109]. In sum, competence frustration and perceptions of self-efficacy may mediate the relation between pain and functional disability. In addition, a potential bidirectional relationship between competence and pain-related outcomes might be observed. That is, pain might impair adolescents' feelings of competence satisfaction, yet at the same time, competence satisfaction (and its support) might foster adaptive living with chronic pain, as is argued below.

#### 4.2.2. A Competence-Supportive Context Is a Moderator: “Feeling Supported in My Competences Helps Me to Pursue Important Goals”

Although adolescents' feelings of competence can be challenged by the presence of pain, a competence-supportive context may be a moderating factor in the relation between pain and functional disability [98]. Youngsters are less inclined to use avoidant mechanisms and are likely to experience less pain-related disability when they feel supported by parents, teachers, or peers in their need for competence, through their engagement in an academic, social, or athletic context [34, 95]. To illustrate, a study by Bursch et al. [108] showed that children from parents who were the most confident about their child's ability to manage pain experienced less somatic symptoms and a better functioning in the child [108]. Thus, parental provision of competence support and their confidence in their child's ability to deal with pain might reduce avoidant behaviors (e.g., towards school or leisure activities) and limit the resulting decline in competences.

Not only parents' but also teacher support may be important. A study of Vervoort et al. [9] showed that teachers' competences support of children with pain improved school attendance despite pain and minimized bullying experiences. Therefore, parents' or teachers' support of the adolescents' competences and self-efficacy may have a moderating role and may constitute a resource to limit avoidant mechanisms, pain-related functional disability, and thus facilitate the pursuit of personal goals.

### 4.3. Relatedness and Chronic Pain

#### 4.3.1. Relatedness as a Mediator: “Living with Pain Makes Me Feel Lonely and Misunderstood”

The third need distinguished within SDT is the need for relatedness, which can also be frustrated by chronic pain. Indeed, adolescents with chronic pain frequently report difficulties in social functioning [110–113]. They often feel different and misunderstood by their peers, partly because living with pain makes them having to carry more responsibilities than other adolescents of their age [96, 111, 114]. For example, they have to know how to deal with medication or to make conscious choices about their activities in order to avoid potential negative consequences (e.g., more pain, more fatigue) [31]. Compared to healthy adolescents, those with chronic pain report less social peer acceptance have fewer friends [99, 104, 115], more often report frustration about their social acceptation [116], and are more likely to suffer from social isolation (Carter et al., 2002); [115, 117]. In addition, they experience more peer victimization and show more fear of rejection than other adolescents [111, 115, 118–120] (Greco et al., 2007; Hjern et al., 2007).

Adolescents with chronic pain are also said to be over-reliant upon their parents for taking care of their social needs because they do not engage in interactions outside the home-setting [46]. For example, children and adolescents often prefer not to spend time with friends when the level of pain is too intense or might become intense during the social event [111]. Moreover, there is an inverse relation between social isolation and the motivation to engage in activities; that is, their isolation caused by pain in turn decreases their motivation to participate in social, leisure, and school activities [5, 7, 99, 101, 106]. In other words, relatedness frustration may explain (i.e., mediate) the relation between chronic pain and social avoidance, which may instigate a downward cycle. That is, avoiding social situations may yield more social isolation and relatedness frustration, further decreasing their motivation to attend social activities.

#### 4.3.2. A Relatedness-Supportive Context as a Moderator: “Good Time Spent with My Friends Distracts Me from My Pain”

As stated above, positive social interactions might be challenged by chronic pain [96, 97, 111], yet, at the same time, they bring important short-term and long-term benefits. For example, Eccleston et al. [5] found that peer relationships and positive social interactions with peers are protective factors in the development of adolescents with chronic pain, minimizing the risk for adolescents to suffer from social isolation. Moreover, positive social interactions had positive consequences for their levels of pain and pain management and it decreased avoidance mechanisms [24, 49]. Further, adolescents described perceived peer support and talking about pain with close friends as a resource, because it was related to better functional ability and better quality of life [26, 107, 114]. Similarly, research on patients with fibromyalgia and rheumatic diseases showed that higher perceived social support predicted fewer adjustment problems and fewer symptoms of depression and anxiety [107, 121]. Not only peers, but also the family context may play an important role in pain-related social avoidance. Indeed, perceived social support from one's family was found to play a moderating role as it relates to more child-reported quality of life [107]. However, when parents restrain activity involvement and peer relationships because of their own pain-related fears and anxiety, it increases pain-related avoidance in the adolescent [16].

To sum up, experiencing social support and having satisfying and positive relationships with friends and family seems to be a protective factor that may decrease the risk of avoidance behaviors. Thus, even though if it is often threatened by pain, experiencing a sense of relatedness may be considered as an important resource for adolescents with chronic pain.

## 5. Research and Clinical Implications

### 5.1. Implications for Future Research

Our topical review discussed how basic need satisfaction is a resilience pathway to adaptive functioning in pediatric pain. A growing amount of literature exists on resilient mechanisms that help people to live adaptively with pain. Optimism, positive emotions, and social and family support are considered as resilience factors [24]. Inspired by previous work [9, 122], we propose that the satisfaction of the needs for autonomy, relatedness, and competence are resilience factors, as well as the parents' and teachers' support of these needs. That is, need satisfaction and experiencing a need-supportive context would facilitate living adaptively with pain and facilitate recovery. On the contrary, when children are frustrated in their needs and when they grow up in a need-thwarting environment, which could be the case when living with chronic pain, they might feel discouraged to attend valued activities and pursue personal goals. Thus, need frustration is considered as a risk factor that might increase functional disability.

In this paper, we joined the forces of two different research fields, that is, the psychological development and the pediatric pain literature, to deepen our comprehension of resources and risk mechanisms when living with chronic pain. SDT may yield interesting insights into dynamics involved in chronic pain, as it highlights the potential protective role of need satisfaction and need-supportive interactional context. This approach is substantially different from other approaches, such as the FAM [12], which primarily has a psychopathological approach to study pain-related functional disability and mainly focuses on risks and maladaptive behaviors. These models have been questioned [21, 123, 124], and new approaches increasingly consider chronic pain as an abnormal situation to which patients respond normally, rather than a normal situation to which people respond maladaptively [21]. Considering people with chronic pain as “normal” involves developing theories that are not (only) based on a psychopathological model but also on models representing normal development. Moreover, SDT also may help to better understand under which conditions parental involvement is helpful and adaptive and when it is problematic (e.g., in the case of overprotection, which is likely to be experienced as need-frustrating).

The above theoretical suggestions concerning the moderating and mediating role of the basic psychological needs should be empirically tested, with quantitative and qualitative methods. According to our knowledge, no studies to date have assessed associations between chronic pain, need satisfaction, and parental need-support, among children or adolescents suffering from chronic pain. In the same vein, the relation between pain and personality traits related to the needs (i.e., controlled, autonomous, and impersonal orientation) [54] should also be explored, as personality traits might influence the way people live their life with chronic pain, as this was shown for various clinical groups (e.g., [125]). The use of existing validated questionnaires assessing basic psychological need satisfaction and frustration, and its contextual support [28] would be welcome in a pediatric pain population. In addition, qualitative research is also desirable in order to gain a more in-depth understanding of the nature of basic need satisfaction and frustration in the context of chronic pain. The way parents deal specifically with their child's chronic pain should be explored, and the consequences of parental practices for child's experiences of need satisfaction and frustration should also be tested. Moreover, some child's factors, as for instance his/her response to pain or his/her personality, might also be controlled, as they could influence parental responses to the child's pain. Finally, observational research (i.e., through the use of videos and interactions coding schemes) also would be relevant in order to avoid self-report bias when assessing the effects of social interactions on the basic psychological needs, pain management and goal pursuit.

### 5.2. Clinical Implications

If empirical research is able to provide support for our hypothesized model, the present framework might have implications for clinical practice. Focusing on adolescents' psychological needs and the pursuit of personal goals might help fostering resilience through the exploration of potential resources for living with pain. Several clinical interventions based on an SDT approach have shown their relevance for various health issues. For example, supporting the patient's autonomy [73, 90], encouraging autonomous motivation to change health behaviors [126, 127], and internalizing a feeling of competence [128] have been found to be helpful for improving treatments outcomes and physical health in diverse clinical groups (e.g., obese children, patients with chronic pain, and tobacco-dependent people) (Ryan et al., 2008). Those resilience factors then could be important levers for pain management, both at the individual and interactional level. Indeed, when parents observe their child in pain, they might start worrying and might be inclined to increase their involvement to help them cope with their pain, paradoxically worsening the adolescents' pain experiences [16, 129, 130]. By contrast, when parents are supportive of their child's psychological needs, they are more likely to alleviate the negative consequence of their pain experiences. Parents can do so by, for instance, being sensitive for their child's valued goals and by helping them to find his/her way to achieve it despite chronic pain. For example relatedness need-supportive parenting may involve supporting the adolescent's wish to spend time with friends despite knowing the risk for the child to hurt and the possible negative consequences (e.g., more fatigue, more pain). On the opposite, overprotective parents might prefer keeping the adolescent home to rest instead of allowing him/her to do sport with friends (or any other personally valued activities) as they may worry that the engagement in such activities would worsen the child's pain symptoms. However, these practices ironically would bring frustration in the need for relatedness and, over the longer term, decrease the adolescent's motivation to attend social activities because he/she might feel rejected from the group of peers.

Those examples show the relevance of integrating therapeutic programs that originate from the developmental literature to improve (1) need satisfaction in youths with chronic pain and (2) parental support of their child's/adolescent's needs. Both might improve pain therapies and treatment outcomes. For example, programs fostering need-supportive parenting (e.g., the “how-to parenting program”; [131–133]) could be adapted and implemented in the context of families with children with chronic pain.

## 6. Conclusion

The developmental context is often neglected in the pediatric pain literature [26], but it could provide important information to understand resilience mechanisms to live adaptively with chronic pain. As discussed in the present paper, Self-Determination Theory may provide a developmental framework that can foster our understanding of why adolescents with chronic pain are likely to adopt avoidant behaviors or, by contrast, to pursue personal goals and live adaptively. Further research into developmental pediatric pain models might improve our theoretical understanding of chronic pain and inform future clinical interventions.

## Figures and Tables

**Table 1:** Definitions and examples of how children's/adolescents' three basic psychological needs can be satisfied or frustrated.

	Needs satisfaction	Needs frustration
Autonomy	Experiencing a sense of volition, personal choice, and psychological freedom in one's actions	Feeling forced or coerced to act in a certain way
Competence	Feeling capable to achieve desired outcomes	Feelings of doubt and failure concerning one's efficacy
Relatedness	Feelings of intimacy and genuine connection with others	Feelings of relational exclusion and loneliness

## References

[1] Perquin C. W., Hazebroek-Kampschreur A. A., Hunfeld J. A. (2000). Pain in children and adolescents: a common experience. *Pain*.

[2] King S., Chambers C. T., Huguet A. (2011). The epidemiology of chronic pain in children and adolescents revisited: a systematic review. *Pain*.

[3] Huguet A., Miró J. (2008). The severity of chronic pediatric pain: an epidemiological study. *Journal of Pain*.

[4] Hunfeld J. A., Perquin C. W., Duivenvoorden H. J. (2001). Chronic pain and its impact on quality of life in adolescents and their families. *Journal of Pediatric Psychology*.

[5] Eccleston C., Wastell S., Crombez G., Jordan A. (2008). Adolescent social development and chronic pain. *European Journal of Pain*.

[6] Fisher E., Palermo T. M. (2016). Goal pursuit in youth with chronic pain. *Children*.

[7] Gauntlett-Gilbert J., Eccleston C. (2007). Disability in adolescents with chronic pain: patterns and predictors across different domains of functioning. *Pain*.

[8] Stommen N. C., Verbunt J. A., Goossens M. E. (2016). Future goals of adolescents and young adults with chronic musculoskeletal pain. *European Journal of Pain*.

[9] Vervoort T., Logan D. E., Goubert L., De Clercq B., Hublet A. (2014). Severity of pediatric pain in relation to school-related functioning and teacher support: an epidemiological study among school-aged children and adolescents. *Pain*.

[10] Ryan R. M., Deci E. L. (2000). Intrinsic and extrinsic motivations: classic definitions and new directions. *Contemporary Educational Psychology*.

[11] Fisher E., Heathcote L. C., Eccleston C., Simons L. E., Palermo T. M. (2017). Assessment of pain anxiety, pain catastrophizing, and fear of pain in children and adolescents with chronic pain: a systematic review and meta-analysis. *Journal of Pediatric Psychology*.

[12] Vlaeyen J. W., Linton S. J. (2000). Fear-avoidance and its consequences in chronic musculoskeletal pain: a state of the art. *Pain*.

[13] Vlaeyen J. W., Linton S. J. (2012). Fear-avoidance model of chronic musculoskeletal pain: 12 years on. *Pain*.

[14] Simons L. E., Kaczynski K. J. (2012). The Fear Avoidance model of chronic pain: examination for pediatric application. *Journal of Pain*.

[15] Asmundson G. J., Noel M., Petter M., Parkerson H. A. (2012). Pediatric fear-avoidance model of chronic pain: Foundation, application and future directions. *Pain Research & Management*.

[16] Goubert L., Simons L. E. (2013). *Cognitive Styles and Processes in Paediatric Pain, Oxford textbook of pediatric pain*.

[17] Vlaeyen J. W. S., Kole-Snijders A. M. J., Boeren R. G. B., van Eek H. (1995). Fear of movement/(re)injury in chronic low back pain and its relation to behavioral performance. *Pain*.

[18] Norton P. J., Asmundson G. J. (2004). Anxiety sensitivity, fear, and avoidance behavior in headache pain. *Pain*.

[19] Simons L. E., Sieberg C. B., Carpino E., Logan D., Berde C. (2011). The fear of pain questionnaire (FOPQ): assessment of pain-related fear among children and adolescents with chronic pain. *The Journal of Pain*.

[20] Simons L. E. (2016). Fear of pain in children and adolescents with neuropathic pain and complex regional pain syndrome. *Pain*.

[21] Crombez G., Eccleston C., Van Damme S., Vlaeyen J. W., Karoly P. (2012). Fear-avoidance model of chronic pain: the next generation. *Clinical Journal of Pain*.

[22] Van Damme S., Kindermans H. (2015). A self-regulation perspective on avoidance and persistence behavior in chronic pain: new theories, new challenges?. *Clinical Journal of Pain*.

[23] Vlaeyen J. W., Crombez G., Linton S. J. (2016). The fear-avoidance model of pain. *Pain*.

[24] Cousins L. A., Kalapurakkel S., Cohen L. L., Simons L. E. (2015). Topical review: resilience resources and mechanisms in pediatric chronic pain. *Journal of Pediatric Psychology*.

[25] Eccleston C, Palermo T. M., Williams A., Lewandowski A., Morley S. (2009). Psychological therapies for the management of chronic and recurrent pain in children and adolescents. *Cochrane Database Systematic Reviews*.

[26] Palermo T. M., Putnam J., Armstrong G., Daily S. (2007). Adolescent autonomy and family functioning are associated with headache-related disability. *Clinical Journal of Pain*.

[27] Vansteenkiste M., Niemiec C. P., Soenens B., Urdan T., Karabenick S. (2010). The development of the five mini-theories of self-determination theory: an historical overview, emerging trends, and future directions. *Advances in Motivation and Achievement: Vol. 16, The Decade Ahead*.

[28] Chen B., Vansteenkiste M., Beyers W. (2015). Basic psychological need satisfaction, need frustration, and need strength across four cultures. *Motivation and Emotion*.

[29] Deci E. L., Ryan R. M. (2000). The “what” and “why” of goal pursuits: human needs and the self-determination of behavior. *Psychological Inquiry*.

[30] Crombez G., Lauwerier E., Goubert L., Van Damme S. (2016). Goal pursuit in individuals with chronic pain: a personal project analysis. *Frontiers in Psychology*.

[31] Jordan A. (2010). Parenting an adolescent with chronic pain: impact on parents and association with adolescent functioning. *Reviews in Pain*.

[32] Palermo T. M., Chambers C. T. (2005). Parent and family factors in pediatric chronic pain and disability: an integrative approach. *Pain*.

[33] Simons L. E., Smith A., Kaczynski K., Basch M. (2015). Living in fear of your child’s pain: the Parent Fear of Pain Questionnaire. *Pain*.

[34] Sinclair C. M., Meredith P., Strong J., Feeney R. (2016). Personal and contextual factors affecting the functional ability of children and adolescents with chronic pain: a systematic review. *Journal of Developmental and Behavioral Pediatrics*.

[35] Vowles K. E., Cohen L. L., McCracken L. M., Eccleston C. (2010). Disentangling the complex relations among caregiver and adolescent responses to adolescent chronic pain. *Pain*.

[36] Vervoort T., Caes L., Trost Z., Notebaert L., Goubert L. (2012). Parental attention to their child’s pain is modulated by threat-value of pain. *Health Psychology*.

[37] Wilson A. C., Lewandowski A. S., Palermo T. M. (2011). Fear-avoidance beliefs and parental responses to pain in adolescents with chronic pain. *Pain Research and Management*.

[38] Fales J. L., Essner B. S., Harris M. A., Palermo T. M. (2014). When helping hurts: miscarried helping in families of youth with chronic pain. *Journal of Pediatric Psychology*.

[39] Joussemet M., Mageau G. A., Koestner R. (2014). Promoting optimal parenting and children’s mental health: a preliminary evaluation of the how-to parenting program. *Journal of Child and Family Studies*.

[40] Sieberg C. B., Williams S., Simons L. E. (2011). Do parent protective responses mediate the relation between parent distress and child functional disability among children with chronic pain?. *Journal of Pediatric psychology*.

[41] DuPen M. M., van Tilburg M. A., Langer S. L., Murphy T. B., Romano J. M., Levy R. L. (2016). Parental protectiveness mediates the association between parent-perceived child self-efficacy and health outcomes in pediatric functional abdominal pain disorder. *Children*.

[42] Janssens K. A., Oldehinkel A. J., Rosmalen J. G. (2009). Parental overprotection predicts the development of functional somatic symptoms in young adolescents. *Journal of Pediatrics*.

[43] Welkom J. S., Hwang W.-T., Guite J. W (2013). Adolescent pain catastrophizing mediates the relationship between protective parental responses to pain and disability over time. *Journal of Pediatric Psychology*.

[44] Jordan A. L., Eccleston C., Osborn M. (2007). Being a parent of the adolescent with complex chronic pain: an interpretative phenomenological analysis. *European Journal of Pain*.

[45] Race D. L., Sims-Gould J., Tucker L. B. (2016). ‘It might hurt, but you have to push through the pain’ Perspectives on physical activity from children with juvenile idiopathic arthritis and their parents. *Journal of Child Health Care*.

[46] Evans S., Meldrum M., Tsao J. C., Fraynt R., Zeltzer L. K. (2010). Associations between parent and child pain and functioning in a pediatric chronic pain sample: a mixed methods approach. *International Journal on Disability and Human Development*.

[47] Langer S. L., Romano J. M., Levy R. L., Walker L. S., Whitehead W. E. (2009). Catastrophizing and parental response to child symptom complaints. *Children’s Health Care*.

[48] Maciver D., Jones D., Nicol M. (2010). Parents’ experiences of caring for a child with chronic pain. *Qualitative Health Research*.

[49] Fisher E., Keogh E., Eccleston C. (2016). Adolescents’ approach-avoidance behaviour in the context of pain. *Pain*.

[50] Masten A. S. (2001). Ordinary magic: resilience processes in development. *American Psychologist*.

[51] Pielech M., Vowles K., Wicksell R. (2017). Acceptance and commitment therapy for pediatric chronic pain: theory and application. *Children*.

[52] Smith A. M., Sieberg C. B., Odell S., Randall E., Simons L. E. (2015). Living life with my child’s pain: the parent pain acceptance questionnaire PPAQ. *Clinical Journal of Pain*.

[53] Wallace D. P., McCracken L. M., Weiss K. E., Harbeck-Weber C. (2015). The role of parent psychological flexibility in relation to adolescent chronic pain: further instrument development. *Journal of Pain*.

[54] Deci E. L., Ryan R. M. (1985). The general causality orientations scale: self-determination in personality. *Journal of Research in Personality*.

[55] Deci E. L., Vansteenkiste M. (2004). Self-determination theory and basic need satisfaction: understanding human development in positive psychology. *Ricerche di Psicologia*.

[56] Vansteenkiste M., Ryan R. M. (2013). On psychological growth and vulnerability: basic psychological need satisfaction and need frustration as a unifying principle. *Journal of Psychotherapy Integration*.

[57] Ryan R. M. (1995). Psychological needs and the facilitation of integrative processes. *Journal of Personality*.

[58] Soenens B., Vansteenkiste M. (2005). Antecedents and outcomes of self-determination in 3 life domains: the role of parents’ and teachers’ autonomy support. *Journal of Youth and Adolescence*.

[59] Britton P. C., VanOrden K. A., Hirsch J. K., Niemiec C. P., Williams G. C. (2014). Basic psychological needs, suicidal ideation, and risk for suicidal behavior in young adults. *Suicide and Life-Threatening Behavior*.

[60] Olafsen A. H., Niemiec C. P., Halvari H., Deci E. L., Williams G. C. (2017). On the dark side of work: a longitudinal analysis using self-determination theory. *European Journal of Work and Organizational Psychology*.

[61] Ryan R. M., Deci E. L., Vansteenkiste M, Cicchetti D. (2016). Autonomy and autonomy disturbances in self‐development and psychopathology: research on motivation, attachment, and clinical process. *Developmental Psychopathology*.

[62] Williams G. C., Halvari H., Niemiec C. P., Sorebo O., Olafsen A. H., Westbye C. (2014). Managerial support for basic psychological needs, somatic symptom burden and work-related correlates: a self-determination theory perspective. *Work and Stress*.

[63] Haerens L., Aelterman N., Vansteenkiste M., Soenens B., Van Petegem S. (2015). Do perceived autonomy-supportive and controlling teaching relate to physical education students’ motivational experiences through unique pathways? Distinguishing between the bright and dark side of motivation. *Psychology of Sport and Exercise*.

[64] Joussemet M., Landry R., Koestner R. (2008). A self-determination theory perspective on parenting. *Canadian Psychology*.

[65] Ahmad I., Vansteenkiste M., Soenens B. (2013). The relations of Arab Jordanian adolescents’ perceived maternal parenting to teacher-rated adjustment and problems: the intervening role of perceived need satisfaction. *Developmental Psychology*.

[66] Soenens B., Vansteenkiste M., Lens W. (2007). Conceptualizing parental autonomy support: adolescent perceptions of promotion of independence versus promotion of volitional functioning. *Developmental Psychology*.

[67] Soenens B., Vansteenkiste M., Sierens E. (2009). How are parental psychological control and Autonomy‐Support related? A Cluster‐Analytic approach. *Journal of Marriage and Family*.

[68] Soenens B., Vansteenkiste M. (2010). A theoretical upgrade of the concept of parental psychological control: proposing new insights on the basis of self-determination theory. *Developmental Review*.

[69] Van Petegem S., Soenens B., Vansteenkiste M., Beyers W. (2015). Rebels with a cause? Adolescent defiance from the perspective of reactance theory and self‐determination theory. *Child Development*.

[70] Vansteenkiste M., Lens W., Deci E. L. (2006). Intrinsic versus extrinsic goal contents in self-determination theory: another look at the quality of academic motivation. *Educational Psychologist*.

[71] Ryan R. M., Patrick H., Deci E. L., Williams G. C. (2008). Facilitating health behaviour change and its maintenance: Interventions based on self-determination theory. *European Psychologist*.

[72] Kindt S., Vansteenkiste M., Loeys T., Goubert L. (2016). Helping motivation and well-being of chronic pain couples: a daily diary study. *Pain*.

[73] Kindt S., Vansteenkiste M., Loeys T. (2015). When is helping your partner with chronic pain a burden? The relation between helping motivation and personal and relational functioning. *Pain Medicine*.

[74] Uysal A., Ascigil E., Turunc G. (2017). Spousal autonomy support, need satisfaction, and well-being in individuals with chronic pain: a longitudinal study. *Journal of Behavioral Medicine*.

[75] Schrooten M. G., Wiech K., Vlaeyen J. W. (2014). When pain meets … pain-related choice behavior and pain perception in different goal conflict situations. *Journal of Pain*.

[76] Van Damme S., Van Ryckeghem D. M., Wyffels F., Van Hulle L., Crombez G. (2012). No pain no gain? Pursuing a competing goal inhibits avoidance behavior. *Pain*.

[77] Zimmer-Gembeck M. J., Collins W. A., Adams G., Beronzky M. D. (2003). Autonomy development during adolescence. *Blackwell Handbook of Adolescence*.

[78] Soenens B., Vansteenkiste M., van Petegem S. (2018). *Autonomy in Adolescent Development, Towards Conceptual Clarity*.

[79] Van Petegem S., Vansteenkiste M., Beyers W. (2013). The jingle–jangle fallacy in adolescent autonomy in the family: in search of an underlying structure. *Journal of Youth and Adolescence*.

[80] Steinberg L. (2002). *Adolescence*.

[81] Qin L., Pomerantz E. M., Wang Q. (2009). Are gains in decision-making autonomy during early adolescence beneficial for emotional functioning? The case of the United States and China. *Child Development*.

[82] Dishion T. J., Nelson S. E., Bullock B. M. (2004). Premature adolescent autonomy: parent disengagement and deviant peer process in the amplification of problem behaviour. *Journal of Adolescence*.

[83] Van Petegem S., Beyers W., Vansteenkiste M., Soenens B. (2012). On the association between adolescent autonomy and psychosocial functioning: examining decisional independence from a self-determination theory perspective. *Developmental Psychology*.

[84] Chen B., Vansteenkiste M., Beyers W., Soenens B., Van Petegem S. (2013). Autonomy in family decision making for Chinese adolescents: disentangling the dual meaning of autonomy. *Journal of Cross-Cultural Psychology*.

[85] Logan D. E., Guite J. W., Sherry D. D., Rose J. B. (2006). Adolescent-parent relationships in the context of adolescent chronic pain conditions. *The Clinical Journal of Pain*.

[86] Palermo T. M. (2012). *Cognitive-Behavioral Therapy for Chronic Pain in Children and Adolescents*.

[87] Palermo T. M., Valrie C. R., Karlson C. (2014). Family and parent influences on pediatric chronic pain: a developmental perspective. *American Psychologist*.

[88] Kindt S., Vansteenkiste M., Cano A., Goubert L. (2017). When is your partner willing to help you? The role of daily goal conflict and perceived gratitude. *Motivation and Emotion*.

[89] Williams G. C., Freedman Z. R., Deci E. L. (1998). Supporting autonomy to motivate patients with diabetes for glucose control. *Diabetes Care*.

[90] Williams G. C., Rodin G. C., Ryan R. M., Grolnick W. S., Deci E. L. (1998). Autonomous regulation and long-term medication adherence in adult outpatients. *Health Psychology*.

[91] Kindt S., Vansteenkiste M., Josephy H., Bernardes S. F., Goubert L. (2018). Helping your partner with chronic pain: the importance of helping motivation, received social support and its timeliness. *Pain Medicine*.

[92] Maharaj S., Daneman D., Olmsted M., Rodin G. (2004). Metabolic Control in Adolescent Girls: Links to relationality and the female sense of self. *Diabetes Care*.

[93] Lewandowski A., Drotar D. (2006). The relationship between parent-reported social support and adherence to medical treatment in families of adolescents with type 1 diabetes. *Journal of Pediatric Psychology*.

[94] Miller V. A., Drotar D. (2003). Discrepancies between mother and adolescent perceptions of diabetes-related decision-making autonomy and their relationship to diabetes-related conflict and adherence to treatment. *Journal of Pediatric Psychology*.

[95] Claar R. L., Walker L. S., Smith C. A. (1999). Functional disability in adolescents and young adults with symptoms of irritable bowel syndrome: the role of academic, social, and athletic competence. *Journal of Pediatric Psychology*.

[96] Forgeron P., McGrath P. (2008). Self-identified needs of youth with chronic pain. *Journal of Pain Management*.

[97] Haraldstad K., Sørum R., Eide H., Natvig G. K., Helseth S. (2011). Pain in children and adolescents: prevalence, impact on daily life, and parents’ perception, a school survey: pain in children and adolescents. *Scandinavian Journal of Caring Sciences*.

[98] Kaczynski K. J., Claar R. L., LeBel A. A. (2012). Relations between pain characteristics, child and parent variables, and school functioning in adolescents with chronic headache: a comparison of tension-type headache and migraine. *Journal of Pediatric Psychology*.

[99] Roth-Isigkeit A., Thyen U., Stöven H., Schwarzenberger J., Schmucker P. (2005). Pain among children and adolescents: restrictions in daily living and triggering factors. *Pediatrics*.

[100] Gorodzinsky A. Y., Hainsworth K. R., Weisman S. J. (2011). School functioning and chronic pain: a review of methods and measures. *Journal of Pediatric Psychology*.

[101] Logan D. E., Simons L. E., Stein M. J., Chastain L. (2008). School impairment in adolescents with chronic pain. *Journal of Pain*.

[102] Sato A. F., Hainsworth K. R., Khan K. A., Ladwig R. J., Weisman S. J., Davies W. H. (2007). School absenteeism in pediatric chronic pain: identifying lessons learned from the general school absenteeism literature. *Children’s Healthcare*.

[103] Konijnenberg A. Y. (2005). Children with unexplained chronic pain: substantial impairment in everyday life. *Archives of Disease in Childhood*.

[104] Merlijn V. P. B. M., Hunfeld J. A. M., van der Wouden J. C., Hazebroek-Kampschreur A. A. J. M., Koes B. W., Passchier J. (2003). Psychosocial factors associated with chronic pain in adolescents. *Pain*.

[105] Simons L. E., Sieberg C. B., Claar R. L. (2012). Anxiety and functional disability in a large sample of children and adolescents with chronic pain. *Pain Research and Management*.

[106] Logan D. E., Simons L. E., Carpino E. A. (2012). Too sick for school? Parent influences on school functioning among children with chronic pain. *Pain*.

[107] Libby C. J., Glenwick D. S. (2010). Protective and exacerbating factors in children and adolescents with fibromyalgia. *Rehabilitation Psychology*.

[108] Bursch B., Tsao J. C., Meldrum M., Zeltzer L. K. (2006). Preliminary validation of a self-efficacy scale for child functioning despite chronic pain child and parent versions. *Pain*.

[109] Martinez-Calderon J., Zamora-Campos C., Navarro-Ledesma S., Luque-Suarez A. (2018). The role of self-efficacy on the prognosis of chronic musculoskeletal pain: a systematic review. *The Journal of Pain*.

[110] Fleischman K. M., Hains A. A., Davies W. H. (2011). Practitioner perceptions of peer relationships in adolescents with chronic pain. *Journal of Child Health Care*.

[111] Forgeron P., Evans J., McGrath P., Stevens B., Finley G. (2013). Living with difference: exploring the social self of adolescents with chronic pain. *Pain Research and Management *.

[112] Campo J. V., Bridge J., Ehmann M. (2004). Recurrent abdominal pain, anxiety, and depression in primary care. *Pediatrics*.

[113] Walker L. S., Smith C. A., Garber J., Van Slyke D. A (1997). Development and validation of the pain response inventory for children. *Psychological Assessment*.

[114] Moulin V., Akre C., Rodondi P. Y., Ambresin A. E., Suris J. C. (2015). A qualitative study of adolescents with medically unexplained symptoms and their parents. Part 1: experiences and impact on daily life. *Journal of Adolescence*.

[115] Forgeron P., King S., Stinson J. N., McGrath P. J., MacDonald A. J., Chambers C. T. (2010). Social functioning and peer relationships in children and adolescents with chronic pain: a systematic review. *Pain Research and Management*.

[116] Massey E. K., Garnefski N., Gebhardt W. A. (2009). Goal frustration, coping and well-being in the context of adolescent headache: a self‐regulation approach. *European Journal of Pain*.

[117] Carter B., Lambrenos K., Thursfield J. (2002). A pain workshop: an approach to eliciting the views of young people with chronic pain. *Journal of Clinical Nursing*.

[118] Kashikar-Zuck S., Lynch A. M., Graham T. B., Swain N. F., Mullen S. M., Noll R. B. (2007). Social functioning and peer relationships of adolescents with juvenile fibromyalgia syndrome. *Arthritis Care and Research*.

[119] Greco L. A., Freeman K. E., Dufton L. (2007). Overt and relational victimization among children with Ffequent abdominal pain: links to social skills, academic functioning, and health service use. *Journal of Pediatric Psychology*.

[120] Hjern A., Alfven G., Östberg V. (2007). School stressors, psychological complaints and psychosomatic pain: School stressors. *Acta Paediatrica*.

[121] von Weiss R. T., Rapoff M. A., Varni J. W. (2002). Daily hassles and social support as predictors of adjustment in children with pediatric rheumatic disease. *Journal of Pediatric Psychology*.

[122] Goubert L., Trompetter H. (2017). Towards a science and practice of resilience in the face of pain. *European Journal of Pain*.

[123] Eccleston C., Crombez G. (2007). Worry and chronic pain: a misdirected problem solving model. *Pain*.

[124] Maujean A., Sterling M. (2017). “De-pathologising” the psychological responses to injury and pain. *Musculoskeletal Science and Practice*.

[125] Strauss J., Ryan R. M. (1987). Autonomy disturbances in subtypes of anorexia nervosa. *Journal of Abnormal Psychology*.

[126] Kasser T., Ryan R. M. (1996). Further examining the American dream: differential correlates of intrinsic and extrinsic goals. *Personality and Social Psychology Bulletin*.

[127] Vansteenkiste M., Simmons J., Braet C., Bachman C., Deci E. L. (2007). *Promoting Maintained Weight Loss through Healthy Lifestyle Changes among Severely Obese Children: An Experimental Test of Self-Determination Theory*.

[128] Patrick H., Williams G. C., Fortier M. (2007). Supporting autonomy in clinical interventions: Toward successful multiple health behavior change.

[129] Caes L., Vervoort T., Eccleston C., Vandenhende M., Goubert L. (2011). Parental catastrophizing about child’s pain and its relationship with activity restriction: the mediating role of parental distress. *Pain*.

[130] Caes L., Vervoort T., Trost Z., Goubert L. (2012). Impact of parental catastrophizing and contextual threat on parents’ emotional and behavioral responses to their child’s pain. *Pain*.

[131] Faber A., Mazlish E. (1980). How to talk so kids will listen: group workshop kit.

[132] Faber A., Mazlish E. (2012). *How to Talk so Kids will Listen and Listen so Kids will Talk*.

[133] Guite J. W., McCue R. L., Sherker J. L., Sherry D. D., Rose J. B. (2011). Relationships among pain, protective parental responses, and disability for adolescents with chronic musculoskeletal pain: the mediating role of pain catastrophizing. *Clinical Journal of Pain*.

